# A Multi-Center Assessment of Nutrient Levels and Foods Provided by Hospital Patient Menus

**DOI:** 10.3390/nu7115466

**Published:** 2015-11-11

**Authors:** Susan Trang, Jackie Fraser, Lori Wilkinson, Katherine Steckham, Heather Oliphant, Heather Fletcher, Roula Tzianetas, JoAnne Arcand

**Affiliations:** 1 Department of Nutritional Sciences, Faculty of Medicine, University of Toronto, 150 College Street, Toronto, ON M5S 3E2, Canada; susan.trang@mail.utoronto.ca; 2 Department of Food and Nutrition Services, Mount Sinai Hospital, 600 University Avenue, Toronto, ON M5G 1X5, Canada; jackie.m.fraser@gmail.com (J.F.); lori.klin@gmail.com (L.W.); k.steckham@gmail.com (K.S.); rtzianetas@hotmail.com (R.T.); 3 Department of Food and Nutrition Services, Hamilton Health Sciences Centre, Chedoke Site, Sanatorium Road, Hamilton, ON L9C 1C4, Canada; oliphant@hhsc.ca; 4 Department of Food and Nutrition Services, St. Michael’s Hospital, 30 Bond Street, Toronto, ON M5B 1W8, Canada; fletcherh@smh.ca; 5 Faculty of Health Sciences, University of Ontario Institute of Technology, 2000 Simcoe St. North Science Building, Rm 3016, Oshawa, ON L1H 7K4, Canada

**Keywords:** malnutrition, food service, energy, protein, hospital menus

## Abstract

Diets of high nutritional quality can aid in the prevention and management of malnutrition in hospitalized patients. This study evaluated the nutritional quality of hospital patient menus. At three large acute care hospitals in Ontario, Canada, 84 standard menus were evaluated, which included regular and carbohydrate-controlled diets and 3000 mg and 2000 mg sodium diets. Mean levels of calories, macronutrients and vitamins and minerals provided were calculated. Comparisons were made with the Dietary Reference Intakes (DRI) and Canada’s Food Guide (CFG) recommendations. Calorie levels ranged from 1281 to 3007 kcal, with 45% of menus below 1600 kcal. Protein ranged from 49 to 159 g (0.9–1.1 g/kg/day). Energy and protein levels were highest in carbohydrate-controlled menus. All regular and carbohydrate-controlled menus provided macronutrients within the Acceptable Macronutrient Distribution Ranges. The proportion of regular diet menus meeting the DRIs: 0% for fiber; 7% for calcium; 57% for vitamin C; and 100% for iron. Compared to CFG recommended servings, 35% met vegetables and fruit and milk and alternatives, 11% met grain products and 8% met meat and alternatives. These data support the need for frequent monitoring and evaluation of menus, food procurement and menu planning policies and for sufficient resources to ensure menu quality.

## 1. Introduction

Malnutrition, a concern in acute care hospitals, is associated with adverse clinical outcomes, including delayed wound healing and increased length of stay, rates of readmission and increased healthcare costs [1]. The prevalence of malnutrition is estimated to be 45% among patients admitted to hospitals in Canada [2]. Malnutrition is caused by increased energy and protein needs associated with acute or chronic illness and can be exacerbated by poor dietary intake [1]. One aspect of addressing malnutrition is careful menu planning in hospitals, which would ensure that menus provide adequate energy, macronutrients, vitamins and minerals and meet the nutritional needs of patients during a hospital stay [3].

Currently, there are no published national accreditation or provincial standards for menu development in Canadian hospitals. Many hospital food service departments typically develop their own criteria for menu planning and report using Canada’s Food Guide [4] and/or Dietary Reference Intakes (DRIs) [5] as benchmarks [6,7,8]. Few other countries have developed hospital nutrition and menu planning guidelines [9,10,11,12,13]. Existing guidelines make recommendations for single nutrients, as well as provide general menu planning guidance, such as menu structure, number of servings from each food group, portion sizes, food preparation and recipe standardization. A select few further break down recommendations for nutritionally-vulnerable and nutritionally well patients [9,10]. At present, there are only a few studies that have evaluated the nutritional quality of hospital patient menus, and there are no known studies in Canada [3,14]. It is unknown if hospitals meet these basic guidelines for healthy eating and if menus are of adequate nutritional quality to meet the increased nutritional needs of acutely ill patients. Therefore, the primary objective was to assess Canadian public policy related to the food provided to hospitalized patients, by reporting on the nutritional quality of patient menus. Specifically, mean energy, macronutrient, vitamin and mineral levels provided by common diet prescriptions (regular, carbohydrate-controlled and sodium-restricted diet menus) at three large acute care hospitals in Ontario, Canada, were calculated. The secondary objective was to determine the proportion of these menus that met the DRI and Canada’s Food Guide (CFG) guidelines. Despite the careful design and scrutiny that hospital menus receive, we hypothesized that nutrient levels would vary greatly due to a lack of standards guiding menu planning.

## 2. Experimental Section

### 2.1. Study Design

This cross-sectional study evaluated the energy, macronutrient and vitamin and mineral composition, as well as the number of CFG servings provided by patient menus at three academic acute care hospitals in Ontario, Canada, between November 2010 and August 2011. A detailed analysis of sodium levels was published previously [15]. The largest patient populations served in the hospitals were general medical, surgery and cardiology; but other patients were served, including rehabilitation, oncology, obstetrics, neurology and psychiatry. Together, the hospitals had a total of 1935 beds. Each site used tray delivery service, as well as rethermalization technology to warm entrees and soups prior to delivery of the meal to the bedside.

Each hospital independently operated the patient food services department and was responsible for menu planning, which was overseen by dietitians and a menu planning committee. Each site also had a seven-day rotational menu. At each hospital, the seven-day rotational menu for the following diet prescriptions was analyzed: regular diet (*n* = 21), carbohydrate-controlled diet (*n* = 21), 3000 mg sodium-restricted diet (*n* = 21) and 2000 mg sodium-restricted diet (*n* = 21) (example in Online Supplementary Figure S1). The 3000 mg (*i.e.*, “no added salt”) and 2000 mg sodium diets are the standard levels of sodium restriction available in hospitals. At the sites investigated, 49% of patients self-selected their menus. This analysis excluded patient self-selected menus, because the menus that patients choose from are very similar to those that are part of the rotational menu cycle. A focus on the “default” menus that make up the standard menu rotation is a focus, because the foods served on these menus are provided to many patients on a frequent basis; whereas patient-selected menus only impact an individual. Such an approach is impactful when evaluating population health or policy within the clinical setting.

### 2.2. Nutritional Analysis

A customized database containing food manufacturer-specific data was created in ESHA Food Processor SQL Version 10.5.2 (ESHA Research, Salem, OR, USA) and used to estimate the calories, vitamins and minerals provided by menus. In-house recipes were analyzed and also included in the customized database. Menus were entered by trained coders blinded to diet prescription. The DRI cut-point method was used to analyze the proportion of menus meeting DRI recommendations, which included the estimated average requirement or the adequate intake, where applicable [16]. The Acceptable Macronutrient Distribution Ranges (AMDR) were also used to assess nutritional adequacy; 10%–35% total energy from protein, 20%–35% from fat and 45%–65% from carbohydrates. Vitamin and mineral levels were compared to DRIs [5,17] for males 51–70 years of age and were only analyzed for menus submitted by two of the three hospital sites.

The analysis of CFG servings was calculated manually using serving size and classification criteria from CFG [4]. For mixed dishes, manufacturer-supplied ingredient lists and recipes were analyzed (example in Online Supplementary Figure S2). When manufacturer data were not available, a comparable recipe from the Canadian Nutrient File was used. Food group composition was compared to CFG recommendations for adult males 51 years of age and older. Recommendations used for comparisons were 7 servings of vegetables and fruit, 7 servings of grain products, 3 servings of milk and alternatives, and 3 servings of meat and alternatives.

### 2.3. Statistical Analysis

Data from all three sites were pooled for the analysis. Continuous variables were presented as means and standard deviations. Categorical variables were presented as frequencies and percentages. Analysis of variance was used to determine if differences existed between diet prescriptions for continuous variables. When the F ratio from the analysis of variance was significant, Scheffe’s *post hoc* test was used to determine pairwise differences. All statistical analyses were performed with SAS Version 9.1 (2006, SAS Institute Inc., Cary, NC, USA). A *p*-value of <0.05 was considered statistically significant.

## 3. Results

Eighty-four (*n* = 84) menus were collected for the four diet prescriptions. Overall, 45% of these provided less than 1600 kcal, 61% provided less than 1700 kcal and 69% provided less than 1800 kcal. Within each diet prescription, energy was highly variable throughout the seven-day rotation. For example, the range of calories provided by the regular diet menus was from 1296 to 3007 kcal (Table 1). Carbohydrate-controlled menus provided a significantly greater amount of calories compared to all other diet prescriptions (1808 ± 175 kcal, *p* = 0.073), although 43% of the carbohydrate-controlled menus still provided less than 1800 kcal (Table 1, Online Supplementary Table S1).

Among the menus, 30% provided less than 60 g of protein, 64% provided less than 70 g and 87% provided less than 80 g (Online Supplementary Table S2). When expressed as g/kg/day, modelled based on a 70 kg adult, menus provided an average 0.9–1.1 g/kg/day. Within a diet prescription, protein content varied greatly during the seven-day rotation. The 3000 mg sodium-restricted menus had the highest variability ranging from 52 to 159 g per day (Table 1). Compared to regular menus, carbohydrate-controlled menus provided significantly higher amounts of protein (63 ± 9 g *vs.* 77 ± 10 g, *p* = 0.019). Protein levels were below 60 g in 43% of standard “default” regular menus and 38% in both 3000 mg and 2000 mg sodium menus (Online Supplementary Table S2). Average protein, fat and carbohydrate levels, as a percentage of total energy, fell within the AMDR for all diet prescriptions (Table 1).

**Table 1:** Nutritional composition of hospital menus.

	Regular Menus		Carbohydrate-Controlled Menus		3000 mg Menus		2000 mg Menus	
	Standard (*n* = 21)	Standard Range	Standard (*n* = 21)	Standard Range	Standard (*n* = 21)	Standard Range	Standard (*n* = 21)	Standard Range
Calories (kcal)	1673 ± 362	1296–3007	1808 ± 175	1516–2067	1600 ± 210	1281–2168	1687 ± 228	1342–2091
Protein (g)	63 ± 9 ^b^	49–86	77 ± 10 ^a^	61–96	66 ± 22	52–159	67 ± 14	51–102
% Total Energy from Protein	18 ± 5	12–31	22 ± 9	14–38	19 ± 5	13–29	18 ± 4	13–28
<10% of kcal [5]	0 (0%)		0 (0%)		0 (0%)		0 (0%)	
Protein (g/kg) ^e^	0.9 ± 0.1 ^b^	0.7–1.2	1.1 ± 0.1 ^a^	0.9–1.4	1.0 ± 0.3	0.7–2.3	1.0 ± 0.2	0.7–1.5
Fat (g)	52 ± 13	30–83	61 ± 8 ^c,d^	48–76	48 ± 13^b^	28–73	50 ± 14^b^	33–74
% Total Energy from Fat	28 ± 3	21–34	30 ± 3 ^c,d^	25–35	26 ± 5^b^	18–34	26 ± 4^b^	20–32
>35% of kcal [5]	0 (0%)		0 (0%)		0 (0%)		0 (0%)	
Carbohydrate (g)	240 ± 60	193–483	242 ± 24	206–295	226 ± 23	191–278	246 ± 26	202–288
% Total Energy from Carbohydrate	57 ± 3 ^b^	52–64	54 ± 3 ^a,d^	47–58	57 ± 6	38–68	59 ± 5^b^	50–68
<45% of kcal [5]	0 (0%)			0 (%)	1 (5%)		0 (0%)	
Sodium (mg)	2896 ± 606 ^b,c,d^	1746–4531	3406 ± 544 ^a,c,d^	2532–4425	2401 ± 389 ^a,b,d^	1755–3487	1504 ± 296 ^a,b,c^	1114–2149
Fiber (g)	17.6 ± 4.2	10.6–24.0	25.1 ± 4.9	18.0–33.3	17.0 ± 4.4	10.6–27.1	19.2 ± 6.6	10.0–34.1
<30g	14 (100)		10 (71)		14 (100%)		13 (93%)	
Cholesterol (mg)	282 ± 133	131–496	333 ± 136	136–498	295 ± 185	111–641	148 ± 80	58–359
Vitamin C (mg)	106 ± 75	11–248	114 ± 60	27–236	106 ± 74	7–214	107 ± 75	21–215
<75 mg	6 (43%)		2 (14%)		6 (43%)		7 (50%)	
Iron (mg)	11.9 ± 4.2	6.3–22.3	12.7 ± 2.9	8.2–17.2	11.7 ± 3.0	7.9–17.7	11.7 ± 2.9	7.6–16.2
<6 mg	0 (0%)		0 (0%)		0 (0%)		0 (0%)	
Calcium (mg)	682 ± 148	469–1002	1086 ±174	845–1375	658 ± 121	512–917	547 ± 145	367–920
<1000 mg	13 (93%)		6 (43%)		14 (100%)		14 (100%)	

Continuous variables expressed as the mean ± standard deviation and categorical variables as n (%). *p* < 0.05 was considered statistically significant. Cut-points used: fiber, adequate intake 30 g (for men 51–70 years); vitamin C, 75 mg (men 51–70 years); iron, 6 mg (men 51–70 years); calcium, 1000 mg (men 51–70 years); ^a–d^ significant differences from standard ^a^ regular; ^b^ carbohydrate-controlled, ^c^ 3000 mg and ^d^ 2000 mg menus, respectively; ^e^ based on a 70 kg adult.

### 3.1. Other Nutrients

The regular and 3000 mg sodium menus, as well as 93% standard 2000 mg sodium menus provided less than 30 g of fiber (Table 1). Carbohydrate-controlled menus provided the most fiber (25.1 ± 4.9 g); however, 71% of menus contained less than the 30 g recommendation (Table 1). Calcium recommendations were least likely to be met with all 3000 mg and 2000 mg sodium menus, and 93% of regular menus and 43% standard carbohydrate-controlled menus provided less than 1000 mg (Table 1).

### 3.2. Food Guide Servings

**Figure 1:**
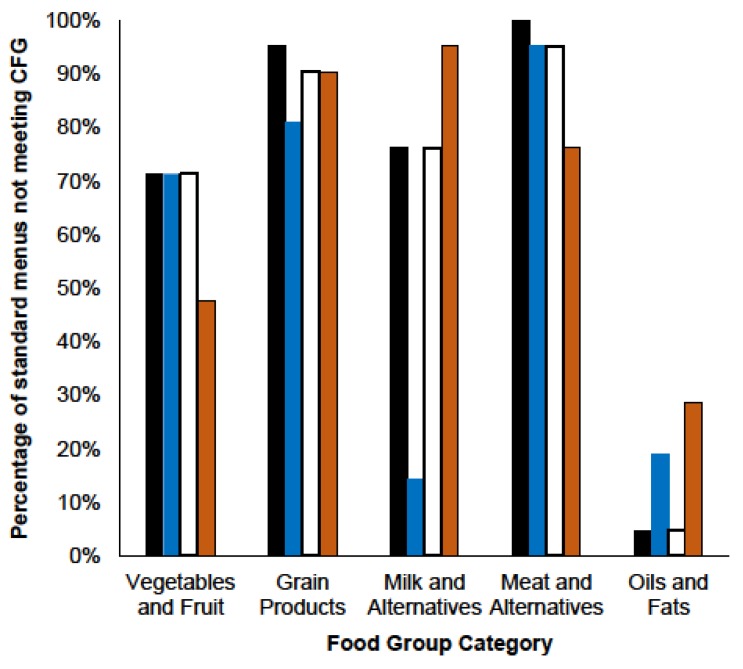
Percentage of standard hospital menus not meeting Canada’s Food Guide recommended number of servings.

There was a large variation in the number of CFG servings provided by the menus. For example, regular menus provided 3.4–8.2 servings of vegetables and fruit (mean 5.9 ± 1.2 servings), 2.3–6.7 servings of grain products (mean 4.6 ± 1.2 servings), 1.1–2.9 servings of milk and alternatives (mean 2.1 ± 0.5 servings) and 0.4–2.9 servings of meat and alternatives (mean 1.7 ± 0.5 servings) (Table 2). Across all diets, only 8% met the recommended number of meat and alternatives; 11% met the recommended number of grain products; and 35% met the recommend number of vegetables and fruit servings and milk and alternative servings (Table 2, Figure 1).

**Table 2:** Food group composition of regular, carbohydrate-controlled, 3000 mg Na and 2000 mg Na standard hospital menus [4].

	Regular Menus		Carbohydrate-Controlled Menus		3000 mg Menus		2000 mg Menus	
	Mean ± SD (*n* = 21)	Range	Mean ± SD (*n* = 21)	Range	Mean ± SD (*n* = 21)	Range	Mean ± SD (*n* = 21)	Range
Vegetables and Fruit	5.9 ± 1.2	3.4–8.2	6.1 ± 1.4	4.6–9.7	5.7 ± 1.3	3.3–8.2	6.7 ± 1.6	3.9–8.7
<7 servings	15 (71%)		15 (71%)		15 (71%)		10 (48%)	
Grain Products	4.6 ± 1.2	2.3–6.7	5.6 ± 0.9	4.1–7.1	4.5 ± 1.4	2.3–7.0	4.6 ± 1.2	2.3–7.1
<7 servings	20 (95%)		17 (81%)		19 (90%)		19 (90%)	
Milk and Alternatives	2.1 ± 0.5	1.1–2.9	2.9 ± 0.5	2.0–3.8	2.0 ± 0.6	0.7–2.9	1.5 ± 0.7	0.6–2.6
<3 servings	16 (76%)		3 (14%)		16 (76%)		20 (95%)	
Meat and Alternatives	1.7 ± 0.5	0.4–2.9	1.7 ± 0.6	0.4–3.3	1.6 ± 0.6	0.4–2.7	1.9 ± 0.6	1.2–3.4
<3 servings	21 (100%)		20 (95%)		20 (95%)		16 (76%)	
Oils and Fats	0.4 ± 0.3	0.2–1.1	0.7 ± 0.3	0.3–1.5	0.4 ± 0.3	0.2–1.1	0.6 ± 0.5	0.2–1.7
>45 mL	1 (5%)		4 (19%)		1 (5%)		6 (29%)	

Continuous variables expressed as the mean ± standard deviation and categorical variables as n (%); vegetables and fruit, grain products, milk and alternatives, meat and alternatives, and oils and fats cut-points were represented as the recommended number of servings for adult males (51+ years).

The number of standard menus for regular (black), carbohydrate-controlled (blue) and 3000 mg (white) and 2000 mg (orange) sodium diet prescriptions that did not meet the recommended number of servings per day of vegetables and fruit (greater than seven), grain products (greater than seven), milk and alternatives (greater than three), meat and alternatives (greater than three) and oils and fats (45 mL or less) outlined in Canada’s Food Guide. Expressed as a percentage of total menus for each diet prescription from all three sites, *n* = 21.

## 4. Discussion

This study provides novel data that evaluated Canadian public policy related to the food provided to hospitalized patients. Menus did not consistently meet recommendations for macronutrient, vitamin and mineral levels or CFG servings, particularly for the protein-rich meat and alternatives food group. The menus under investigation were among the most commonly prescribed in hospitals; therefore, the implications of such findings are broad. The variability and shortfalls observed are not surprising given that there are no published national accreditation or provincial standards to guide calorie, macronutrient or food group composition of hospital menus in Canada. Given the importance of menu quality in reducing the risk of and treating malnutrition, this is an area where leadership is needed to maximize menu quality, including the development of standards and potentially the allocation of resources to support the inclusion of healthy, nutrient-dense foods.

This study found that almost half of the standard regular menus contained less than 1600 kcal/day with energy content varying from 1296 to 3007 kcal/day. Despite careful planning, calorie levels at the lower end of this range are likely insufficient to meet patients’ nutritional requirements, especially considering increased nutritional needs during illness and recovery (e.g., stress, fever, wound healing), where estimated energy requirements can range from 1750 to 2450 kcal/day (25–35 kcal/kg based on a 70 kg person) [18]. At the higher end of the caloric range, providing excessive amounts of food increases food waste and costs. These findings are similar to others. One study in Switzerland reported large variations in calories in patient menus with a mean level of 1981 ± 454 kcal [14]. Another study compared hospital menus to the German Nutrition Society general dietary recommendations and found that mean energy was 1561 kcal, 16% lower than recommended [3].

The lower calorie content of standard menus was related to the lower absolute levels of protein provided by these menus. The mean protein level provided by regular menus was 63 ± 9 g (0.90 g/kg/day based on a 70 kg person), however, at the lower end, provided as little as 49 g/day (0.70 g/kg/day based on a 70 kg person), which would not be sufficient to meet the protein needs of most hospitalized patients who may have elevated protein requirements of 1.0–2.0 g/kg/day [18]. Low protein levels have been reported in other hospital settings. Iff *et al.* (2008) [3] reported a mean protein content of 67 g in menus (0.96 g/kg/day based on a 70 kg person), which would fall below the recommendations for patients undergoing minor or major surgery (1.0–1.2 g/kg/day) [18]. Although Thibault *et al.* (2011) [14] found that mean protein in menus was sufficient for meeting patients’ needs, the amount of protein provided among these menus varied considerably (83 ± 22 g), but on average, was much higher than that observed in the present study.

None of the standard menus consistently met CFG recommendations, and particularly concerning was the relatively low number of protein-rich meat and alternative foods provided (1.7 ± 0.5 servings/day), explaining the low protein levels observed. Also a source of protein and calcium, milk and alternatives recommendations were met in only 35% of standard menus, with an overall average of 2.1 servings/day. While only 35% of standard menus met the CFG recommendations for vegetables and fruit, this would be expected, since menu planning in the hospital typically focuses on providing calorically-dense foods. These findings highlight the inherent inconsistency that the food intake pattern recommended by CFG has in meeting specific nutrient needs [19] and support the need for setting food and nutrient standards appropriate for hospitalized patients, so that food service departments have accepted benchmarks to guide menu planning. In developing these standards, it would be important to consider the nutritional goals of patient populations, rather than directly translating population health guidelines to the hospital setting. Additionally, sufficient budgetary resources would need to be in place to purchase food of high nutritional quality, which has been shown to increase the nutritional quality of hospital menus [20].

A number of hospital nutrition standards have been established in the United States, United Kingdom and Australia [9,10,11,12,13,21]. All established standards within these countries include guidelines for total energy, as well as macronutrient and micronutrient levels, although there is even significant country-to-country variance in the recommended menu nutritional composition. There are no accreditation standards or best practice guidelines for hospital menu planning. In the absence of national or provincial standards, many Canadian hospitals plan menus guided by CFG and DRIs [6,7,8], and some also integrate specific cultural food guides (*i.e.*, Northwest Territories) [22]. Recently, several provinces have developed policies regarding specific nutrients (*i.e.*, sodium and trans fat) [23,24,25]; however, comprehensive nutrition standards that include other key nutrients and whole foods for acute care facilities are lacking, particularly for nutrients concerning malnutrition, such as protein and calories.

There are limitations to our study. First, we compared nutrient levels to the DRIs and food group servings to CFG for males 51 years of age and older, which are both intended for healthy populations and may be inappropriate in clinical settings where patients have altered and/or elevated nutritional requirements. However, these were used in the absence of standards for hospitalized patients, and they reflect typical practice in Canadian foods service departments. Second, this study evaluated menus and not actual food intake. Such an approach is appropriate, since we are evaluating nutrition policy, rather than assessing direct patient nutritional needs. It is likely that our estimates have underreported the degree of nutritional inadequacy, since other studies have reported patient consumption to be as little as 33% of a given meal [1,3,14]. Finally, we relied on manufacturer-provided data for the nutritional analysis under the assumption that the data provided were accurate. Although more accurate, chemical analysis was unfeasible, as our database contained over 700 individual foods and recipes.

## 5. Conclusions

This study demonstrated that patient menus did not consistently meet recommendations for nutrient levels and food servings; instead, menus varied greatly in nutritional content, and a relatively large proportion provided lower levels of calories and protein. These data point to the need for the implementation of frequent monitoring and evaluation of hospital menus, the development of standards and benchmarks to guide hospital menu development and budgetary resources to both support these practices and to procure high-quality foods. Additionally, appropriate nutritional targets for energy, macronutrient and vitamin and mineral levels are needed to ensure that menus are of high nutritional quality in order to address the needs of acute care patients and to optimize patient outcomes.

## Supplementary Materials

Supplementary materials can be accessed at: http://www.mdpi.com/2072-6643/7/11/5466/s1.
