# Supplementary material for: A Multi-Center Assessment of Nutrient Levels and Foods Provided by Hospital Patient Menus
Source: Nutrients. 2015 Nov 11;7(11):9256–64. doi: 10.3390/nu7115466 (PMC4663594; doi:10.3390/nu7115466)
Supplement: Supplementary file 1 [file nutrients-07-05466-s001.docx]

**Supplementary Materials: A Multi-Center Assessment of Nutrient Levels and Foods Provided by Hospital Patient Menus**

Susan Trang, Jackie Fraser, Lori Wilkinson, Katherine Steckham, Heather Oliphant,
Heather Fletcher, Roula Tzianetas and JoAnne Arcand

**Table S1.** Caloric distribution of regular, carbohydrate-controlled, 3000 mg Na and 2000 mg Na standard menus.

| **Calories (g)** | **Regular  (*n* =21)** | **Diabetic  (*n* = 21)** | **3000 mg Na (*n* = 21)** | **2000 mg Na  (*n* =21)** | **Total  (*N* = 84)** |
| --- | --- | --- | --- | --- | --- |
| ≤1299 | 1 (5%) | 0 (0%) | 2 (10%) | 0 (0%) | 3 (4%) |
| 1300–1399 | 3 (14%) | 0 (0%) | 1 (5%) | 1 (5%) | 5 (6%) |
| 1400–1499 | 0 (0%) | 0 (0%) | 3 (14%) | 3 (14%) | 6 (7%) |
| 1500–1599 | 7 (33%) | 4 (19%) | 6 (29%) | 7 (33%) | 24 (29%) |
| 1600–1699 | 4 (19%) | 2 (10%) | 4 (19%) | 3 (14%) | 13 (15%) |
| 1700–1799 | 2 (10%) | 3 (14%) | 2 (10%) | 0 (0%) | 7 (8%) |
| 1800–1899 | 1 (5%) | 6 (29%) | 2 (10%) | 1 (5%) | 10 (12%) |
| 1900–1999 | 1 (5%) | 2 (10%) | 0 (0%) | 4 (19%) | 7 (8%) |
| 2000–2099 | 1 (5%) | 4 (19%) | 0 (0%) | 2 (10%) | 7 (8%) |
| 2100–2199 | 0 (0%) | 0 (0%) | 1 (5%) | 0 (0%) | 1 (1%) |
| 2200–2299 | 0 (0%) | 0 (0%) | 0 (0%) | 0 (0%) | 0 (0%) |
| 2300–2399 | 0 (0%) | 0 (0%) | 0 (0%) | 0 (0%) | 0 (0%) |
| 2400–2499 | 0 (0%) | 0 (0%) | 0 (0%) | 0 (0%) | 0 (0%) |
| ≥2500 | 1 (5%) | 0 (0%) | 0 (0%) | 0 (0%) | 1 (1%) |

Categorical variables expressed as *n* (%).

**Table S2.** Protein distribution of regular, carbohydrate-controlled, 3000 mg Na and 2000 Na mg standard menus.

| **Protein (g)** | **Regular  (*n* = 21)** | **Diabetic  (*n* = 21)** | **3000 mg Na  (*n* = 21)** | **2000 mg Na  (*n* = 21)** | **Total  (*N* = 84)** |
| --- | --- | --- | --- | --- | --- |
| <49 | 1 (5%) | 0 (0%) | 0 (0%) | 0 (0%) | 1 (1%) |
| 50–59 | 8 (38%) | 0 (0%) | 8 (38%) | 8 (38%) | 24 (29%) |
| 60–69 | 8 (38%) | 6 (29%) | 10 (48%) | 5 (24%) | 29 (35%) |
| 70–79 | 3 (14%) | 9 (43%) | 2 (10%) | 5 (24%) | 19 (23%) |
| 80–89 | 1 (5%) | 4 (19%) | 0 (0%) | 1 (5%) | 6 (7%) |
| 90–99 | 0 (0%) | 2 (10%) | 0 (0%) | 0 (0%) | 2 (2%) |
| 100–109 | 0 (0%) | 0 (0%) | 0 (0%) | 2 (10%) | 2 (2%) |
| >110 | 0 (0%) | 0 (0%) | 1 (5%) | 0 (0%) | 1 (1%) |

Categorical variables expressed as *n* (%).
